# Systematic review of the efficacy of brief intervention in reducing alcohol use in women

**DOI:** 10.1186/1940-0640-7-S1-A49

**Published:** 2012-10-09

**Authors:** Carla Gebara, Fernanda Bhona, Aline Vaz, Mayla Diniz, Lelio Lourenço, Ana R Noto

**Affiliations:** 1 School of Medicine, Federal University of São Paulo, São Paulo, Brazil; 2 Department of Social Psychology and Public Health, Federal University of Juiz de Fora, Juiz de Fora, Brazil; 3 Department of Psychology, Federal University of Juiz de Fora, Juiz de Fora, Brazil; 4 Department of Psychobiology, Paulista School of Medicine, São Paulo, São Paulo, Brazil

## 

The aim of this study was to systematically review the efficacy of brief intervention (BI) in reducing alcohol use in women. We performed an electronic search across all databases using Thomson Reuters (formerly ISI) Web of Knowledge to identify multidisciplinary content. The term "brief intervention" was associated with the words "alcohol" and "women." We selected studies published between 2006-2010. Of the 95 studies found, 52 were excluded because they were presented at meetings or did not focus specifically on the subject of interest. Forty-three articles with the central theme of realization and/or evaluation of BI efficacy were included in the analysis. Abstracts of these papers underwent content analysis. The year 2007 had the most publications (13) as did the following journals: *Journal of Studies on Alcohol and Drugs* (4), *Alcoholism: Clinical and Experimental Research* (3), and *Addictive Behaviors* (3). The authors who published most frequently on BI efficacy were "Carey KB,” "Fleming MF,” "Floyd RL,” “Martens MP,” "Nilsen P,” "Saitz R," and "Shang G,” with two publications each. The quantitative approach was prevalent among studies, with randomized controlled trials the most frequent (35). The BIs were conducted mainly in clinical and health-services settings (18). However, interventions were also conducted via the Internet (5) and by telephone (4). Regarding the study population, 22 articles addressed BI for men and women, 14 addressed BI for women, and 9 addressed BI for pregnant women. The efficacy of BI in the reduction of alcohol consumption was confirmed in most research, but some studies showed a reduction only in men. Further specific research using a study population solely comprised of women is deemed necessary.

